# Arrhythmogenic effects of acute electronic cigarette compared to tobacco cigarette smoking in people living with HIV

**DOI:** 10.14814/phy2.16158

**Published:** 2024-07-23

**Authors:** Isabelle Ruedisueli, Katie Shi, Samuel Lopez, Jeffrey Gornbein, Holly R. Middlekauff

**Affiliations:** ^1^ Department of Medicine, Division of Cardiology UCLA David Geffen School of Medicine Los Angeles California USA; ^2^ Departments of Medicine and Computational Medicine UCLA David Geffen School of Medicine Los Angeles California USA

**Keywords:** electronic cigarettes, heart rate variablity, tobacco, nicotine, parasympathetic nerve activity, sympathetic nerve activity

## Abstract

The leading cause of death in people living with HIV (PLWH) is cardiovascular disease, and the high prevalence of tobacco cigarette (TC) smoking is a major contributor. Switching to electronic cigarettes (ECs) has been promoted as a harm reduction strategy. We sought to determine if acute EC compared to TC smoking had less harmful effects on arrhythmogenic risk factors including acute changes in hemodynamics, heart rate variability (HRV), and ventricular repolarization (VR). In PLWH who smoke, changes in hemodynamics, HRV, and VR were compared pre/post acutely using an EC, TC, or puffing on an empty straw on different days in random order, in a crossover study. Thirty‐seven PLWH (36 males, mean age 40.5 ± 9.1 years) participated. Plasma nicotine was greater after TC versus EC use (10.12 ± 0.96 vs. 6.18 ± 0.99 ng/mL, respectively, *p* = 0.004). HR increased significantly, and similarly, after acute EC and TC smoking compared to control. Changes in HRV that confer increased cardiac risk (LF/HF ratio) were significantly smaller after acute EC versus TC use, consistent with a harm reduction effect. In a post‐hoc analysis of PLWH with and without positive concurrent recreational drug use as indicated by point of care urine toxicology testing, this differential effect was only seen in PLWH not currently using recreational drugs. Changes in VR were not different among the three exposures. In PLWH who smoke, EC compared to TC smoking resulted in smaller adverse changes in HRV. This differential effect was accompanied by a smaller increase in plasma nicotine, and was negated by concurrent recreational drug use. Additional studies are warranted in this vulnerable population disproportionately affected by tobacco‐related health disparities.

## 1 INTRODUCTION

The leading cause of death in people living with HIV infection (PLWH) in the United States is cardiovascular disease, largely attributable to premature atherosclerosis (Farahani et al., 2017; Feinstein et al., 2019; Freiberg & So‐Armah, 2016; Marin et al., 2009). The mechanisms underlying this increased cardiovascular disease risk in PLWH are debated and likely include the HIV milieu, consisting of HIV itself, chronic immune dysregulation, and the metabolic effects of anti‐retroviral therapies (ART), as well as traditional cardiac risk factors, including tobacco cigarette (TC) smoking. PLWH are disproportionately affected by tobacco‐related health disparities, with a prevalence of current TC smoking over twice that of the United States adult population (Mdodo et al., 2015). Smoking prevalence in Americans living with HIV approaches 50%, and it has been reported that PLWH lose more life years to smoking‐related diseases, including but not limited to cardiovascular diseases, than to HIV (Feinstein et al., 2019; Helleberg et al., 2013; Tseng et al., 2012). Further, PLWH who smoke are less likely to quit compared to the general population (Cioe et al., 2018; Frazier et al., 2018).

In PLWH, the majority of deaths from cardiovascular disease, in fact up to 86%, are sudden and unexpected, and most are likely attributable to lethal ventricular arrhythmias (Tseng et al., 2012). Smoking may increase risk of sudden cardiac death through its adverse effects on ventricular repolarization (VR) rendering PLWH more vulnerable to ventricular arrhythmias (Dilaveris et al., 2001; Heravi et al., 2019; Ilgenli et al., 2015; Tasolar et al., 2014). Smoking is also associated with autonomic imbalance as measured by decreased heart rate variability, another risk factor of ventricular arrhythmias and sudden cardiac death (McIntosh et al., 2017; Middlekauff et al., 2014). Finally, in PLWH who smoke, acute sympathetic excitation associated with acute smoking may lead to increased myocardial oxygen demand (increased heart rate and blood pressure), as well as, potentially, decreased myocardial oxygen delivery (vasospasm), resulting in supply—demand mismatch, and promoting arrhythmogenesis (Benowitz & Burbank, 2016).

Switching to electronic cigarettes (ECs), which deliver comparable levels of nicotine accompanied by lower levels of non‐nicotine toxicants and carcinogens compared to combusted TCs, has been proposed as a harm reduction approach in vulnerable populations with high smoking prevalence, such as PLWH, who are unable or unwilling to stop smoking (Cioe et al., 2020; Vuong et al., 2023). It is important to examine the evidence that forms the foundation of this harm reduction proposal, that is, that ECs, while not harmless, are less harmful than TCs. Emissions from ECs deliver lower levels of non‐nicotine toxicants compared to TCs (Reilly et al., 2019; Shahab et al., 2017), and it is these non‐nicotine toxicants that likely drive premature atherosclerosis (Csordas & Bernhard, 2013; Messner & Bernhard, 2014). Our group has reported that in the general adult population, acute or chronic EC use compared to TC smoking has smaller adverse effects on endothelial function (Haptonstall et al., 2020), vascular inflammation (Boas et al., 2017), and immune activation and oxidative stress (Kelesidis et al., 2020, 2021a, 2021b), all of which are known to contribute to atherosclerotic cardiovascular disease. However, nicotine is sympathomimetic, and acute and chronic increases in sympathetic tone are widely recognized as potentially arrhythmogenic and may trigger ischemia. Scant data exists supporting the notion that during acute use, ECs are less sympathomimetic or arrhythmogenic compared to TCs, and in fact data are emerging that challenges this premise (Carll et al., 2022; Nguyen et al., 2024).

Accordingly, in this study, we compared the acute hemodynamic, HRV, and VR effects of a fourth generation EC device in PLWH who smoke TCs. We had initially intended to exclude PLWH who smoke who were also concurrently using recreational drugs, reasoning that the potentially larger adverse impact of recreational drug use on these primary outcomes may obscure any benefit of switching from TCs to ECs. The purpose of the study was to test the hypothesis that acute EC use compared with acute TC smoking in PLWH who smoke would have significantly lower adverse effects on changes in hemodynamics, HRV, and VR, known cardiovascular risk factors.

## 2 MATERIALS AND METHODS

The data that support the findings of this study are available from the corresponding author upon reasonable request.

### 2.1 Study population

The study population consisted of male and female PLWH who smoke combusted TCs (self‐reported current, daily TC smoking for at least 1‐year) between the ages of 21 and 60 years. Inclusion criteria included self‐report of chronic HIV infection, ART, with a viral RNA < 50 copies/ml, and CD4^+^ T cell count >500 cells/mm^3^. Exclusion criteria included concurrent active infection and known cardiovascular disease, including non‐sinus rhythm, coronary artery disease, cardiomyopathy, or history of heart failure. EC use was not an exclusion criterion. The experimental protocol was approved by the Institutional Review Board at the University of California, Los Angeles (UCLA), and written informed consent was obtained from each participant. This study was registered on ClinicalTrials.gov with the unique identifier: NCT04568395. H.R.M. had full access to all the data in the study and takes responsibility for its integrity and the data analysis.

### 2.2 Study design

#### 2.2.1 Randomized crossover study

Participants underwent three experimental sessions in random order on different days: (1) straw‐control, (2) EC with 5% nicotine, or (3) commercially available TC.

### 2.3 Exposures

The straw‐control used in the study was a plastic soda straw. Since there is tremendous variability among EC liquids, including flavorings, solvents, nicotine chemical formulation, nicotine concentration, and efficiency of nicotine delivery, as well as variability among device characteristics, including the heating parameters, capacity for varying puff sizes, and the quality of workmanship, we determined that it was imperative to provide the same EC exposure to each participant. Accordingly, we used the JUUL with a standard 5% menthol pod using a standard puffing protocol (detailed below). Conversely, there is much less variability among commercial cigarettes in terms of the nicotine delivery, and the non‐nicotine constituents in smoke. Accordingly, we did not provide commercial cigarettes to the PLWH who habitually smoked. Instead, the participant smoked one of their own commercial TCs.

### 2.4 Nicotine and cotinine plasma levels

Immediately before and after each exposure, blood was drawn and sent to the UCLA Clinical Laboratory for measurement of plasma nicotine (half‐life 1 h) and cotinine levels (half‐life 36 h). The assays for plasma nicotine and cotinine were performed by the commercial laboratory, ARUP Laboratories, using the method of quantitative liquid chromatography—tandem mass spectrometry.

### 2.5 Urine toxicology screen

At the beginning of each experiment session, participants provided a urine sample for immediate urine toxicology point of care testing for up to 12 drugs (Alere iCup Dx Pro 2, Avantar).

### 2.6 ECG recording analysis

#### 2.6.1 Heart rate variability

An electrocardiogram (ECG) was recorded for 5 min at baseline and immediately after the acute exposure in the supine position. Five‐minute ECG recordings were used for analysis using commercially available software (LabChart 8, AD Instruments) for HRV in the frequency domain and time domain as previously described (Shaffer & Ginsberg, 2017). Briefly, in the frequency domain, HRV, analyzed in normalized units to correct for differences in total power, is divided into three components: low frequency (LFnu, 0.004–0.15 Hz) largely indicative of sympathetic activity and/or baroreflex function, high frequency (HFnu, 0.15–0.4 Hz) indicative of cardiac vagal nerve activity, and the LF‐to‐HF ratio (LF/HF), indicative of overall sympathetic to vagal balance (Shaffer & Ginsberg, 2017). In the time domain analysis, the standard deviation of RR intervals (SDRR) and the root mean square of successive RR interval differences (RMSSD), both of which decrease with a decrease in relative vagal dominance (Shaffer & Ginsberg, 2017), were reported.

#### 2.6.2 Ventricular repolarization

Twelve‐lead ECG recordings were analyzed using commercially available software (Labchart Pro 8 with ECG module, AdInstruments) as previously described (Ruedisueli et al., 2022). For each of the 12 leads, all beats were averaged via block averaging resulting in one PQRST complex per lead for analysis. In the 5‐min recording, approximately three hundred beats were analyzed. The ECG Analysis Module software automatically identified the onset of the QRS complex, the peak of the T wave, and the end of the T wave; cursors were placed on each auto‐identified point and placement was confirmed by at least two investigators (I.R., K.S.). The software designated the Tp‐e interval as the difference between the peak of the T‐wave and the end of the T‐wave. The software automatically identified the end of the T‐wave as the intersection of the tangent to the T‐wave's downslope and the isoelectric line (Panikkath et al., 2011). For inverted T‐waves, the Tp‐e was measured as the interval from the nadir of the T‐wave to the end of the T‐wave (Antzelevitch, 2007). Leads in which T waves were low amplitude (<1.5 mm) or flattened were not included in the analysis (Rautaharju et al., 2009). U‐waves were not included in the Tp‐e interval (Panikkath et al., 2011). QTc was calculated using Bazett's formula (Rautaharju et al., 2009). QT Dispersion (QTD) was calculated as the difference between the maximum QT and minimal QT intervals among the 12 ECG leads.

#### 2.6.3 Blood pressure and heart rate

Systolic blood pressure (SBP), diastolic BP (DBP), mean blood pressure (MAP), and heart rate (HR), were measured after a 10‐min rest period in the supine position at baseline, and after a 5‐min rest period following each exposure, with a noninvasive BP monitor (Casmed 740, Avante Health Solutions and the Capsule Smart Linx Vitals Plus) according to American Heart Association guidelines (Muntner et al., 2019). The same approach to BP measurement was followed at each experimental session pre−/post‐exposure, including sham (straw‐control) smoking.

#### 2.6.4 Experimental session

Studies were conducted between 8 am and 2 pm to avoid the potential influence of circadian rhythm on autonomic tone. Participants were instructed to abstain from smoking, ingesting caffeine, or exercising for at least 12 h before their visit. Participants were also asked to abstain from eating or drinking (except water) 6 h before their visit. As previously described (Nguyen et al., 2024), during the visit, participants were in a supine position in a quiet, temperature‐controlled (21°C) room in the Human Physiology Laboratory at the UCLA Clinical and Translational Research Center. Participants provided a urine sample at the beginning of the study. Skin was cleaned with alcohol wipes and 10 electrodes (3M Red Dot™) were placed on the chest according to standard ECG protocol. Recording electrodes were foam silver‐silver chloride conductors, 3.0 cm in diameter, with adhesive hydrogel. The participant was instrumented, blood was drawn, and after a 10‐min rest period, blood pressure and heart rate were measured. Participants were not allowed to use their cell phones or talk during data acquisition. An ECG was then recorded for 5 min for later analysis for HRV and VR. The ECG electrodes were then detached, and the participant was led down a short corridor to a smoking patio, where they underwent their assigned exposure (straw‐control, EC, or TC). A uniform vaping protocol was enforced to ensure a similar “dose” among interventions as previously described (Nguyen et al., 2024). Participants were instructed to take a 3‐s puff every 30 s for up to 15 min when given a straw or EC. If the participants used a TC, they were instructed to finish the entirety of the cigarette (usually within 10 min). Immediately after vaping or smoking, the participant returned to the examination room, was repositioned in the supine position, and after a 5‐min rest period, heart rate and blood pressure were measured. An ECG was then recorded for 5 min, blood was drawn, and the study was concluded. Investigators were not blinded to type of exposure during the experimental session but were blinded to exposure during the analysis of HRV and VR, which was performed later.

## 3 STATISTICAL METHODS

Primary outcomes were HRV: LFnu, HFnu, LF/HF; VR: Tp‐e, Tpe/QT and Tp‐e/QTc; and hemodynamics: HR. Secondary outcomes were HRV: SDRR, RMSSD; VR: QT, QTc, QTD; and hemodynamics: SBP, DBP, MAP.

Of note, variables are measured at two timepoints during every experimental session: Pre‐exposure and post‐exposure. There are three experimental sessions, each one involving a different exposure (TC, EC, and straw‐control) in random order. It is the change pre/post each exposure that is reported and compared among the three exposures.

For the study population, demographic characteristics, cotinine levels, and baseline hemodynamic, VR, and HRV outcomes were calculated from the first experimental session. The *p* values for comparing continuous variables among groups at (visit 1) baseline were computed using the one‐way analysis of variance (ANOVA) model if the data followed the normal distribution or with the non‐parametric Kruskal–Wallis method otherwise.

Normal quantile plots (not shown) indicate that the post‐pre change values for hemodynamic, HRV, and VR outcomes followed a normal distribution except for an occasional outlier. Mean comparisons across exposures were compared using a robust repeated measure model for a 3 × 3 crossover design controlling for (adjusting for) possible visit effects. Robust methods were used to automatically down‐weight outliers. Robust means are reported. The overall Wald test across exposures was computed under the robust repeated measure model for a given outcome (e.g., for Tp‐e). Pairwise robust mean comparisons between one exposure and another within a group were not considered statistically significant if the corresponding overall test was not significant (generalization of the Fisher least significant difference criterion). Using this criterion for significance reduces the probability of false positive (type I) errors.

The rank‐based Spearman correlation was computed to assess the association between changes in nicotine versus changes in heart rate and HRV parameters.

The repeated measure model for this crossover design uses the correlations across time in the same participants to adjust the estimated mean profiles and their corresponding standard errors. Using this model, observations on participants with partial data (i.e., only 1 or 2 visits) can be included with little or no bias.

### 3.1 Comparison of subgroups with positive or negative current recreational drug use

When it became apparent that a large proportion of PLWH who smoke that were screened for our study were also using recreational drugs, we soon recognized the public health urgency to include this group. Further, our study design offered a unique opportunity to compare the acute effects of TCs and ECs in a heterogeneous group, since each individual served as their own control. Accordingly, PLWH who smoke TCs who concomitantly use recreational drugs were also enrolled. In this secondary, post hoc analysis, PLWH were grouped according to positive or negative current recreational drug use verified by results from urine toxicology screen. The same statistical approach described above was employed for comparing continuous hemodynamic, VR, and HRV post/pre change to determine differences among exposures (TC, EC, and straw control) within each subgroup, stratified by recreational drug use, controlling for visit.

## 4 RESULTS

### 4.1 Study population

Thirty‐seven PLWH participated in the study. Demographics and baseline hemodynamics, HRV, and VR parameters are displayed on Table 1. ART included bictegravir‐emtricitabine‐tenofovir alafenamide (*n* = 22), emtricitabine‐rilpivirine‐tenofovir alafenamide (*n* = 3), emtricitabine‐tenofovir alafenamide (*n* = 3), abacavir‐dolutegravir‐lamivudine (*n* = 3), elvitegravir‐cobicistat‐emtricitabine‐tenofovir alafenamide (*n* = 2), and other (*n* = 4). Only one of the 37 participants was female, despite our outreach efforts. Not all participants completed all 3 visits although all 37 participants had at least one visit. One participant was missing straw and TC, one participant was missing EC and TC, one participant was missing straw and EC. One participant was missing EC only.

**TABLE 1:** Study population characteristics.

	*n* = 37
Mean age (years)	40.5 ± 9.1
Sex (male/female)	36/1
Cotinine (ng/mL)	96.1 ± 111.1
Race/Ethnicity	
African American	11
Caucasian	14
Hispanic	11
Unknown	1
BMI (kg/m^2^)	26.3 ± 5.6
Hemodynamics	
Heart rate (bpm)	71.3 ± 12.2
SBP (mmHg)	116.2 ± 10.4
DBP (mmHg)	73.0 ± 9.8
MAP (mmHg)	86.6 ± 9.3
Heart rate variability	
LF (nu)	51.7 ± 21.5
HF (nu)	44.4 ± 18.6
LF/HF	1.87 ± 2.16
SDRR (ms)	44.7 ± 30.2
RMSSD (ms)	45.1 ± 48.3
Ventricular repolarization	
Tp‐e (ms)	87.0 ± 16.0
Tp‐e/QT	237.0 ± 37.0
Tp‐e/QTc	220.0 ± 35.0
QT (ms)	410.0 ± 57.0
QTc (ms)	441.0 ± 48.0
QTD (ms)	80.8 ± 49.0

*Note*: Values ± SEM.

Abbreviations: bpm, beats per minute; DBP, diastolic blood pressure; HF, high frequency; LF, low frequency; MAP, mean arterial pressure; nu, normalized units; QTD, QT Dispersion; RMSSD, root mean square of successive RR interval differences; SBP, systolic blood pressure; SDRR, standard deviation of RR intervals; Tp‐e, Tpeak‐end.

### 4.2 Increase in plasma nicotine following acute exposures

The acute rise in plasma nicotine was significantly greater after acute TC smoking compared to acute EC use: (10.12 ± 0.96 vs. 6.18 ± 0.99 ng/mL respectively, *p* = 0.004), and both were significantly greater compared to straw control (−0.55 ± 1.04 ng/mL, each comparison, *p* < 0.00001).

### 4.3 Changes in hemodynamics following acute exposures (Figure 1 and Table 2)

**FIGURE 1:**
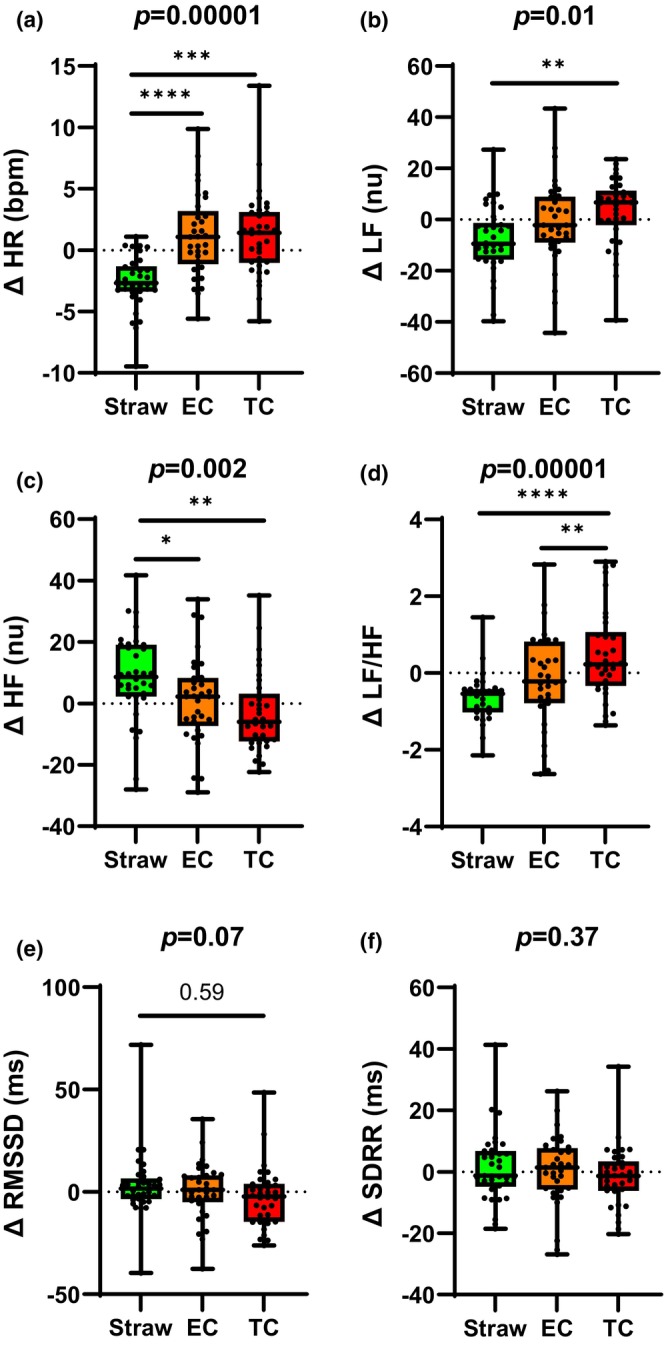
Acute electronic versus tobacco cigarette smoking effects on HR and heart rate variability. (a) HR increased similarly and significantly after acute EC and TC smoking compared to control. In the frequency domain, the increase in sympathetic tone and decrease in vagal tone as estimated by changes in LFnu (b) and HFnu (c) respectively were significantly greater after acute TC smoking compared to control and similar to acute EC use. However, the adverse overall shift in autonomic balance towards sympathetic predominance (LF/HF ratio, d) was significantly less after acute EC versus TC use. There was a trend towards a greater decrease in vagal tone after acute TC smoking compared to control and acute EC use as estimated by RMSSD but not SDRR in the time domain (e and f, respectively). bpm, beats per minute; EC, electronic cigarette; HFnu, high frequency; LFnu, low frequency; nu, normalized units; RMSSD, root mean square of successive RR interval differences; SDRR, standard deviation of RR intervals; TC, tobacco cigarette. *p < 0.05, **p < 0.01, ***p < 0.001, ****p < 0.0001. Error bars are SEM.

**TABLE 2:** PLWH exposure effects.

	Straw‐control	EC	TC	Overall *p* value
	*n* = 35	*n* = 34	*n* = 35	
Hemodynamics				
Δ SBP (mmHg)	4.8 ± 1.8	7.8 ± 1.7	8.5 ± 1.6	0.25
Δ DBP (mmHg)	2.2 ± 1.2	4.4 ± 1.1	4.3 ± 1.1	0.32
Δ MAP (mmHg)	3.0 ± 1.3	4.7 ± 1.3	5.0 ± 1.2	0.40
Ventricular repolarization				
Δ Tp‐e (ms)	9.6 ± 1.3	7.0 ± 1.3	6.2 ± 1.2	0.46
Δ Tp‐e/QT	21.4 ± 3.3	19.8 ± 3.0	19.6 ± 2.9	0.76
Δ Tp‐e/QTc	22.5 ± 3.0	16.0 ± 2.8	15.0 ± 2.7	0.21
Δ QT (ms)	14.4 ± 2.1	9.3 ± 2.0	2.5 ± 2.0	0.064
Δ QTc (ms)	12.3 ± 2.8	12.4 ± 2.7	10.1 ± 2.6	0.97
Δ QTD (ms)	2.9 ± 2.3	−1.2 ± 1.0	−1.0 ± 2.8	0.19

*Note*: Values ± SEM.

Abbreviations: DBP, diastolic blood pressure; EC, electronic cigarette; MAP, mean arterial pressure; PLWH, people living with HIV; QTD, QT dispersion; SBP, systolic blood pressure; TC, tobacco cigarette; Tp‐e, Tpeak‐end.

Heart rate significantly increased after acute TC smoking and acute EC use compared to the straw‐control (Figure 1a). However, there was no difference in the increase in HR between acute TC smoking and acute EC use. The increases in SBP, DBP, and MAP were not significantly different after acute TC smoking or acute EC use compared to straw‐control (Table 2).

### 4.4 Changes in ECG indices of HRV following acute exposures (Figure 1b–f)

**FIGURE 2:**
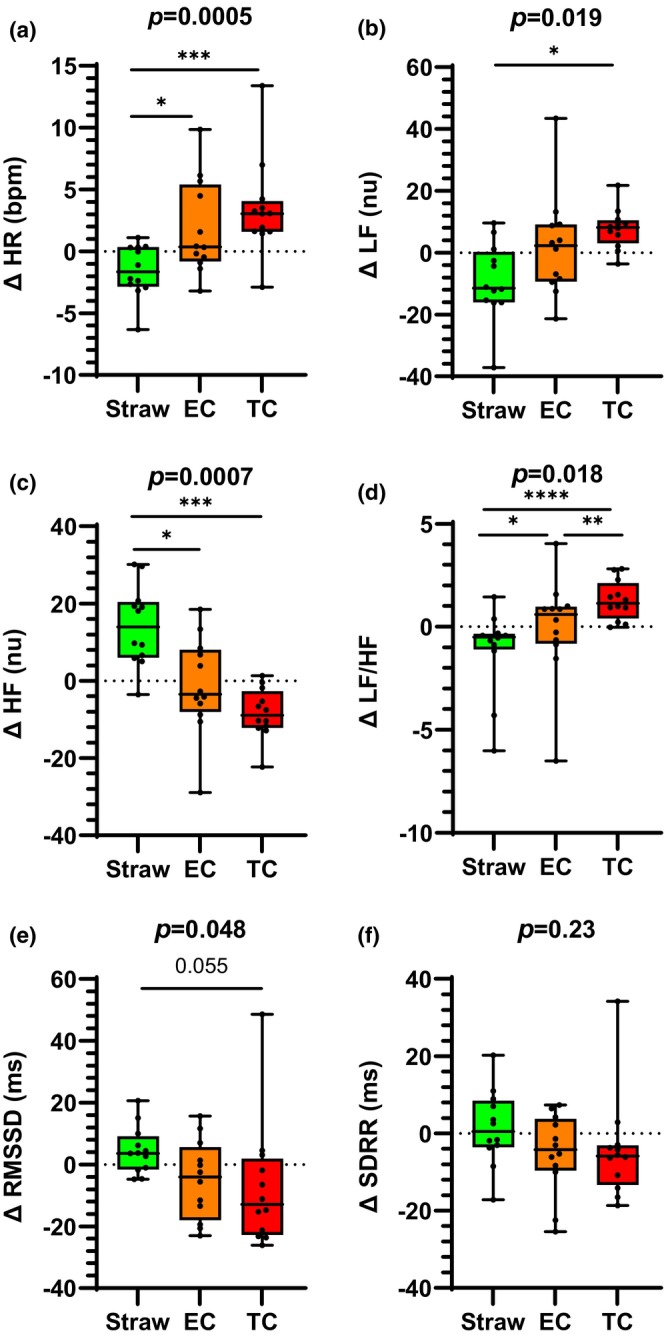
Acute EC versus TC smoking effects on HR and HRV in PLWH not currently using recreational drugs (PLWH‐No Drug). (a) In the PLWH‐No Drug subgroup, HR increased similarly and significantly after acute EC and TC smoking compared to control. In the frequency domain, the increase in sympathetic tone and decrease in vagal tone as estimated by changes in LFnu (b) and HFnu (c) respectively were significantly greater after acute TC smoking compared to control and similar to acute EC use. However, the adverse overall shift in autonomic balance towards sympathetic predominance (LF/HF ratio, d) was significantly less after acute EC versus TC use. Vagal tone tended to decrease after acute TC smoking compared to control but this decrease was not different from acute EC use as estimated by RMSSD (e). The decreases in vagal tone as estimated by SDRR after EC and TC use was not different from control (f). bpm, beats per minute; EC, electronic cigarette; HFnu, high frequency; LFnu, low frequency; nu, normalized units; RMSSD, root mean square of successive RR interval differences; SDRR, standard deviation of RR intervals; TC, tobacco cigarette. *p < 0.05, **p < 0.01, ***p < 0.001, ****p < 0.0001. Error bars are SEM.

There were significant differences following acute TC, EC, and straw use in the change in HRV parameters when measured in the frequency domain. In the frequency domain, LFnu increased significantly after smoking the TC compared to straw‐control; in fact, LFnu actually decreased after using the straw control (Figure 1b). However, the change in LFnu after acutely using the EC was not statistically different from the change after smoking the TC, or from the straw control. HFnu decreased significantly after acute TC smoking and after acute EC use compared to straw control (Figure 1c). However, there was no difference in the decrease in HFnu between acute TC smoking and acute EC use. Finally, after acute TC smoking and after acute EC use, LF/HF increased significantly (Figure 1d). Importantly, this increase in LF/HF was significantly greater after smoking the TC compared to using EC (Figure 1d). For HRV in the time domain, RMSSD tended to decrease after acute TC smoking but did not reach statistical significance (Figure 2e). The change in RMSSD was not significantly different after acute TC smoking and acute EC use. The changes in SDRR were not different among the 3 exposures (Figure 1f).

### 4.5 Changes in ECG indices of ventricular repolarization following acute exposures (Table 2)

There were no significant differences in any of the primary indices of ventricular repolarization, Tp‐e, Tp‐e/QT, and Tp‐e/QTc, after acute TC smoking or acute EC use compared to the straw‐control (Table 2). Interestingly, the change in the secondary outcome, QT, tended to be greater after the TC compared to straw control, although it did not reach significance. This trend in the shortening of the QT after TC smoking may be explained by the increase in heart rate since there was no difference when QT was corrected for HR (QTc; Bazett's). There was no difference in the change in QTD among the 3 exposures (Table 2).

### 4.6 Correlation between change in HR and HRV vs. change in plasma nicotine (Table 3)

**TABLE 3:** Correlation of change in plasma nicotine with changes in heart rate and heart rate variability.

	Spearman correlation	*p* value
Δ Heart rate (bpm)	0.57	0.0001
Δ LF (nu)	0.22	0.031
Δ HF (nu)	−0.23	0.018
Δ LF/HF	0.22	0.026
Δ SDRR (ms)	−0.12	0.220
Δ RMSSD (ms)	−0.17	0.081

Abbreviations: bpm, beats per minute; HF, high frequency; LF, low frequency; nu, normalized units; RMSSD, root mean square of successive RR interval differences; SDRR, standard deviation of RR intervals.

The increase in heart rate was strongly correlated with the increase in plasma nicotine. The changes in HRV in the frequency domain were weakly correlated with the change in plasma nicotine (Table 3).

### 4.7 Sub‐group analysis according to current recreational drug use

During patient accrual and confirmed during data analysis, it became evident that there were potentially two distinct sub‐groups enrolled: PLWH with positive urine toxicology tests (PLWH‐Drug, *n* = 25), and PLWH with negative urine toxicology tests (PLWH‐No Drug, *n* = 12). Drugs included psychomotor stimulants (*n* = 19), cannabis (*n* = 16), benzodiazepines (*n* = 7), and opiates (*n* = 2) with most (*n* = 19) participants testing positive for more than one drug. To determine if current recreational drug use impacted the potential arrhythmogenic effects of TCs or ECs as estimated by hemodynamics, HRV and VR, a subgroup analysis was performed.

### 4.8 Baseline characteristics in PLWH with and without current recreational drug use (Table 4)

**TABLE 4:** Study population with and without recreational drug use baseline characteristics.

	PLWH‐drug	PLWH‐No drug	*p* value
	*n* = 25	*n* = 12	
Mean age (years)	39.6 ± 10.1	42.3 ± 6.9	0.27
Sex (M/F)	25/0	11/1	0.32
Cotinine (ng/mL)	100.8 ± 21.5	86.8 ± 36.4	0.24
Race			0.69
African American	6	5	
Caucasian	11	3	
Hispanic	7	4	
Unknown	1	0	
BMI (kg/m^2^)	25.7 ± 5.7	27.4 ± 5.5	0.31
Hemodynamics			
Heart rate (bpm)	72.2 ± 13.7	69.3 ± 8.7	0.66
SBP (mmHg)	116.0 ± 11.4	116.5 ± 8.6	0.56
DBP (mmHg)	71.6 ± 10.0	76.2 ± 9.0	0.18
MAP (mmHg)	85.5 ± 9.8	89.0 ± 8.2	0.27
Heart rate variability			
LF (nu)	52.60 ± 18.22	68.02 ± 19.40	0.07
HF (nu)	45.06 ± 14.68	31.73 ± 15.07	0.03
LF/HF	1.14 ± 1.52	2.14 ± 2.41	0.03
SDRR (ms)	36.00 ± 25.60	36.70 ± 16.94	0.96
RMSSD (ms)	25.61 ± 28.79	25.04 ± 16.14	0.62
Ventricular repolarization			
Tp‐e (ms)	87.0 ± 9.0	87.0 ± 19.0	0.62
Tp‐e/QT	240.0 ± 42.0	234.0 ± 25.0	0.86
Tp‐e/QTc	220.0 ± 41.0	218.0 ± 20.0	0.93
QT (ms)	409.0 ± 68.0	410.0 ± 25.0	0.22
QTc (ms)	442.0 ± 56.0	438.0 ± 23.0	0.60
QTD (ms)	86.3 ± 57.0	69.4 ± 27.0	0.38

*Note*: Values ± SEM.

Abbreviations: bpm, beats per minute; DBP, diastolic blood pressure; HF, high frequency; LF, low frequency; MAP, mean arterial pressure; nu, normalized units; PLWH, people living with HIV; QTD, QT Dispersion; RMSSD, root mean square of successive RR interval differences; SBP, systolic blood pressure; SDRR, standard deviation of RR intervals; Tp‐e, Tpeak‐end.

There were no significant differences in participant characteristics, baseline plasma cotinine levels, baseline hemodynamics or VR parameters between groups with (PLWH‐Drug) and without (PLWH‐No Drug) current recreational drug use (Table 4). However, in PLWH‐Drug versus PLWH‐No Drug, HRV parameters measured in the frequency domain were significantly different between the groups. PLWH‐Drug had higher resting HFnu, and lower LF/HF. LFnu tended to be lower in the PLWH‐Drug group as well (Table 4).

### 4.9 PLWH with negative urine drug toxicology screen (PLWH‐No drug) subgroup

#### 4.9.1 Increase in plasma nicotine following acute exposures in the PLWH‐No drug subgroup

The acute rise in plasma nicotine was significantly greater after acute TC smoking compared to acute EC use: (11.88 ± 1.57 vs. 4.41 ± 1.53 ng/mL respectively, *p* = 0.0004), and both were significantly greater compared to straw control (−0.14 ± 1.53 ng/mL, each comparison, *p* < 0.05).

#### 4.9.2 Changes in hemodynamics, HRV and VR following acute exposures in the PLWH‐No drug subgroup (Figure 2 and Table 5)

**TABLE 5:** PLWH‐No drug: exposure effects.

	Straw‐control	EC	TC	Overall *p* value
	*n* = 12	*n* = 12	*n* = 12	
Hemodynamics				
Δ SBP (mmHg)	7.1 ± 2.6	7.7 ± 2.6	9.8 ± 2.7	0.50
Δ DBP (mmHg)	2.3 ± 1.7	3.9 ± 1.7	4.5 ± 1.8	0.57
Δ MAP (mmHg)	4.0 ± 1.8	4.1 ± 2.0	4.5 ± 2.0	0.81
Ventricular repolarization				
Δ Tp‐e (ms)	8.8 ± 2.0	5.1 ± 2.0	5.8 ± 2.0	0.54
Δ Tp‐e/QT	21.2 ± 4.7	15.1 ± 4.7	20.5 ± 4.8	0.75
Δ Tp‐e/QTc	16.4 ± 4.4	6.5 ± 4.4	10.9 ± 4.5	0.35
Δ QT (ms)	12.1 ± 3.1	7.3 ± 3.1	1.5 ± 3.2	0.10
Δ QTc (ms)	14.2 ± 4.1	9.0 ± 4.1	10.0 ± 4.3	0.87
Δ QTD (ms)	1.9 ± 3.3	−2.6 ± 3.3	0.4 ± 3.4	0.69

*Note*: Values ± SEM.

Abbreviations: DBP, diastolic blood pressure; EC, electronic cigarette; MAP, mean arterial pressure; PLWH, people living with HIV; QTD, QT Dispersion; SBP, systolic blood pressure; TC, tobacco cigarette; Tp‐e, Tpeak‐end.

Heart rate significantly increased after acute TC smoking and acute EC use compared to the straw‐control (Figure 2a). There was no difference in the increase in HR after acute TC smoking compared to acute EC use. There were no differences in SBP, DBP, and MAP among the three exposures (Table 5).

The changes in HRV in the time and frequency domains were consistent with a statistically significant shift towards lower HFnu, RMSSD, SDRR, and higher LFnu after acute TC smoking and after acute EC use compared to straw‐control (Figure 2b–f). Importantly, acute TC smoking was associated with a significantly greater increase in LF/HF compared to acute EC use (Figure 2d). There were no differences in any of the VR parameters among the three exposures (Table 5).

### 4.10 PLWH with positive urine drug toxicology screen (PLWH‐drug) subgroup

#### 4.10.1 Increase in plasma nicotine following acute exposures in the PLWH‐drug subgroup

The acute rise in plasma nicotine was not different after acute TC smoking compared to acute EC use: (8.35 ± 1.12 vs. 7.95 ± 1.42 ng/mL respectively, *p* = 0.96), and both were significantly greater compared to straw control (−0.97 ± 1.53 ng/mL, each comparison, *p* < 0.00001).

#### 4.10.2 Changes in hemodynamics, HRV and VR following acute exposures in the PLWH‐Drug subgroup (Table 6)

**TABLE 6:** PLWH‐Drug subgroup: exposure effects.

	Straw‐control	EC	TC	Overall *p* value
	*n* = 22	*n* = 21	*n* = 22	
Hemodynamics				
Δ SBP (mmHg)	2.6 ± 2.4	7.9 ± 2.1	7.1 ± 1.9	0.035
Δ DBP (mmHg)	2.0 ± 1.7	5.0 ± 1.4	4.2 ± 1.3	0.029
Δ MAP (mmHg)	2.0 ± 1.8	5.4 ± 1.6	5.5 ± 1.4	0.030
Ventricular repolarization				
Δ Tp‐e (ms)	10.5 ± 1.7	9.0 ± 1.6	6.6 ± 1.4	0.55
Δ Tp‐e/QT	21.5 ± 4.3	24.5 ± 3.8	18.7 ± 3.4	0.57
Δ Tp‐e/QTc	23.5 ± 4.0	20.8 ± 3.5	15.5 ± 3.1	0.22
Δ QT (ms)	16.6 ± 2.9	11.3 ± 2.6	6.4 ± 2.2	0.14
Δ QTc (ms)	9.7 ± 3.8	13.1 ± 3.4	12.2 ± 3.0	0.94
Δ QTD (ms)	2.3 ± 3.1	−0.9 ± 2.7	−2.7 ± 2.4	0.059

*Note*: Values ± SEM.

Abbreviations: DBP, diastolic blood pressure; EC, electronic cigarette; MAP, mean arterial pressure; PLWH, people living with HIV; QTD, QT Dispersion; SBP, systolic blood pressure; TC, tobacco cigarette; Tp‐e, Tpeak‐end.

**FIGURE 3:**
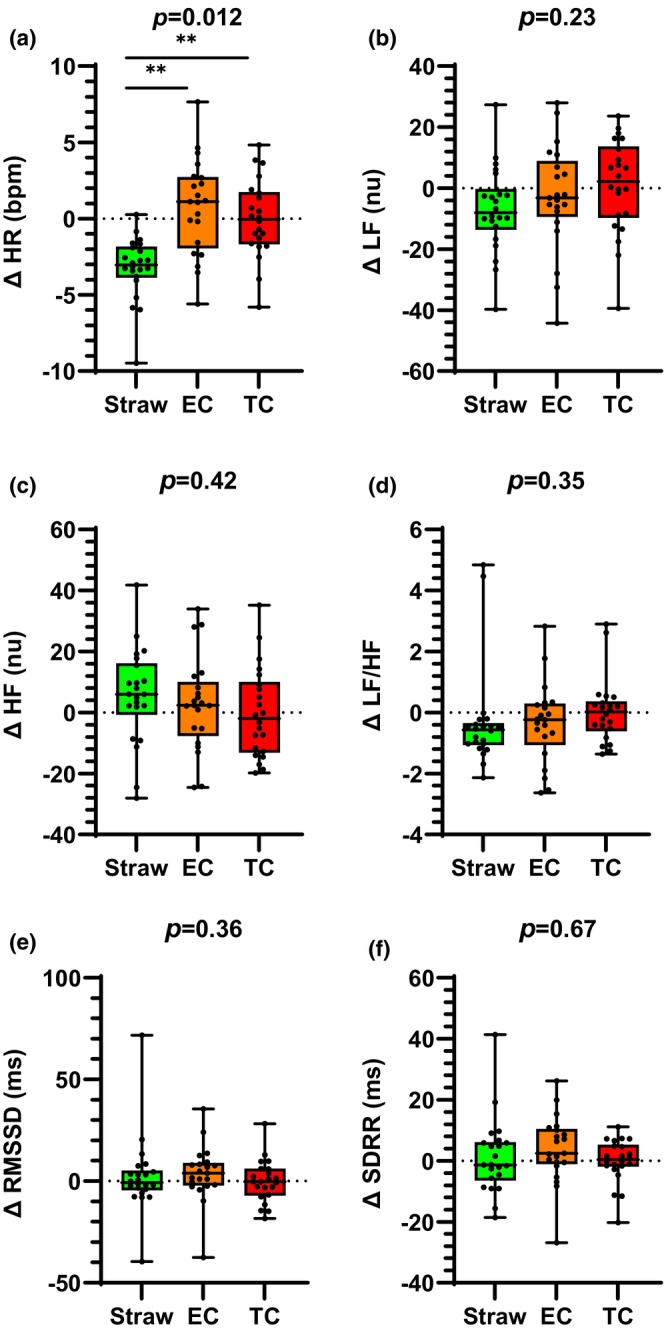
Acute EC versus TC smoking effects on HR and HRV in PLWH currently using recreational drugs (PLWH‐Drug). (a) In the PLWH‐Drug subgroup, HR increased similarly and significantly after acute EC and TC smoking compared to control. There were no differences between acute TC use and EC use on autonomic nerve activity as estimated by HRV parameters in the frequency domain (b–d) or the time domain (e and f). bpm, beats per minute; EC, electronic cigarette; HFnu, high frequency; LFnu, low frequency; nu, normalized units; RMSSD, root mean square of successive RR interval differences; SDRR, standard deviation of RR intervals; TC, tobacco cigarette. **p < 0.01. Error bars are SEM.

Heart rate significantly increased after acute TC smoking and acute EC use compared to the straw‐control (Figure 3a). There was no difference in the increase in HR following acute TC smoking compared to acute EC use. Similarly, SBP, DBP, and MAP increased after acute TC smoking, but these increases were similar to those following acute EC use (Table 6). In contrast to the PLWH‐No Drug subgroup, in the PLWH‐Drug subgroup, the changes in HRV parameters were not different among the three acute exposures (Figure 3b–f). Similarly, there were no differences in any of the changes in VR parameters among the three exposures (Table 6).

## 5 DISCUSSION

To our knowledge, this is the first study in PLWH to investigate the acute, potentially arrhythmogenic, changes associated with smoking a TC compared to using an EC. The major new findings in this study are that (1) the acute hemodynamic effects, including increases in heart rate and blood pressure are not significantly different following acutely smoking a TC compared to using an EC; (2) acute changes in the HRV variables HF and LF were not significantly different following acutely smoking a TC compared to using an EC. However, overall sympathetic predominance, as estimated by LF/HF, is greater following acute TC smoking compared to acute EC use. This finding must be understood with the caveat that although plasma nicotine levels increased significantly after each exposure, the increase was greater after TC smoking compared to EC use. (3) Acute changes in VR were not significantly different following acutely smoking a TC compared to using an EC; and finally, (4) acute changes in hemodynamics and HRV following acute TC smoking and EC use were related to changes in plasma nicotine. Taken collectively, these findings further the notion that ECs, although not harmless, compared to TCs may have significantly less harmful acute, potentially arrhythmogenic, effects.

Recognized 60 years ago by the Surgeon General to increase mortality, including cardiac mortality (Schroeder & Koh, 2014), TCs remain the most prevalent modifiable risk factor for cardiovascular disease, including sudden death, in the United States today. The mechanisms whereby TCs increase cardiovascular mortality include not only their potent chronic atherogenic effects (Csordas & Bernhard, 2013; Messner & Bernhard, 2014), but also their role as an acute trigger for adverse cardiac events. In fact, upon smoking cessation, cardiovascular risk, including sudden death risk, declines precipitously in the first‐year post‐quitting, reaching that of a non‐smoker after approximately 15–20 years (Holford et al., 2014; Sandhu et al., 2012). One mechanism whereby smoking is believed to trigger acute cardiovascular events is through nicotine's strong acute sympathomimetic effect (Haass & Kubler, 1997; Middlekauff et al., 2014). Acute sympathetic excitation increases myocardial oxygen demand through increases in HR and BP, leading to ischemia, and potentially precipitating arrhythmias (Benowitz & Burbank, 2016). Although early reports of the hemodynamic effects of acute EC use compared to TC smoking were consistent with significantly lower acute increases in HR and BP (Garcia et al., 2020), we have recently reported that, in the general population, hemodynamic responses to the fourth generation ECs are not different from those of TCs (Nguyen et al., 2024). The findings from the current study expand those findings to PLWH.

Changes in HRV reflecting changes in autonomic balance, including decreased HF, RMSSD, and SDRR and increased LF and LF/HF have been associated with increased cardiovascular risk (Dekker et al., 2000; Hillebrand et al., 2013; Kleiger et al., 1987; Liao et al., 2002; Tsuji et al., 1996). In the earliest studies, decreased HRV was found to confer increased cardiac risk in the post‐myocardial infarction population (Kleiger et al., 1987). Follow‐up studies have reported that decreased HRV was associated with increased cardiovascular risk in patients with coronary artery disease, diabetes, and importantly, even populations without known cardiovascular disease (Dekker et al., 2000; Hillebrand et al., 2013; Liao et al., 2002; Tsuji et al., 1996). We have recently reported in never smokers, that acute EC use is associated with this same pattern of autonomic imbalance that was strongly associated with increased cardiovascular risk (Moheimani, Bhetraratana, Peters, et al., 2017). However, in the general population that smokes, we have reported that acute EC use (Arastoo et al., 2020), even acute use of the fourth generation pod device (Nguyen et al., 2024), was not associated with these same adverse changes in HRV. Importantly, the findings in the current study, conducted in PLWH, are starkly different from those in the general population. In the current study, acute TC smoking *was* associated with a change in HRV associated with increased cardiac risk. The effects of acute EC use also were associated with similar, but not identical, changes in HRV. Adverse effects on overall autonomic balance as represented by LF/HF were significantly less after using the EC compared to smoking the TC.

Although this finding in PLWH of a significantly smaller adverse effect of ECs compared to TCs on autonomic balance is consistent with the notion that ECs are less harmful than TCs, this occurred in the setting of a smaller increase in plasma nicotine following the EC exposure protocol compared to smoking a single TC, and in this and prior studies, changes in HRV are correlated with changes in plasma nicotine. Several important points are worth emphasizing: First, although the increase in plasma nicotine was smaller following acute EC use compared to TC smoking, the increase was nonetheless significant. Second, the difference in nicotine levels occurred in PLWH despite using the same protocol we have used in many studies for many years, which, in the general adult population, reliably and consistently produced similar plasma nicotine levels as smoking one TC (Arastoo et al., 2020; Haptonstall et al., 2020; Nguyen et al., 2024; Ruedisueli et al., 2023). Potential explanations the difference in the current study include a possible interaction of HIV drugs or the HIV milieu that impacted metabolism of nicotine delivered by the EC device differently from the TC. Alternatively, some or most of our participants may had minimal familiarity or experience with ECs, which may have contributed to the disparity, since puffing on an EC is reportedly quite different from puffing on a TC (Talih et al., 2015; Wagener et al., 2021). There is a learning curve to using an EC (Lee et al., 2015; Wagener et al., 2021). The third and final point to emphasize is that ad libitum vaping behavior differs from ad libitum smoking behavior (St Helen et al., 2016). By the nature of a combusted cigarette, which once lit, will continue to burn, people tend to “bolus‐dose” TC emissions. Conversely, an EC can be used intermittently throughout the day, since emissions are only released in response to a puff; accordingly, people tend to “intermittent‐dose” EC emissions. In ad libitum use of ECs, the rise in plasma nicotine was more gradual than that reported in people who smoke TCs (St Helen et al., 2016). Therefore, it is not inconceivable that the smaller increase in plasma nicotine in this acute exposure study is not unlike the potentially smaller and/or more gradual acute increases in nicotine and potentially, in sympathetic nerve activity, in people who use ECs.

Interestingly, following the straw control session, during which participants puffed on a straw every 30 s for up to 15 min, the same protocol that was used during EC use, changes in HRV and HR reflected an increase in vagal tone. There are several possible explanations for this finding. First, controlled breathing (e.g., breathing at 12 breaths per minute) has been shown to be associated with an increase in vagal tone as measured by HRV (Barutcu et al., 2005; Driscoll & Dicicco, 2000; Moheimani, Bhetraratana, Yin, et al., 2017). However, this increase in vagal tone was measured *during* controlled breathing, not after. How long this effect may persist after the cessation of controlled breathing was not studied and is unknown. Additionally, during this “time control” experimental session, it is possible that the participant simply became more relaxed and comfortable with us over the course of the experiment. Or finally, it is possible that participants who had abstained from smoking the morning of the experimental session were experiencing some withdrawal symptoms, and perhaps puffing on the straw, which simulated the act of smoking, provided some relief. Whatever the explanation, this finding reinforces the importance of including a control session in acute exposure studies.

Changes in VR have been reported in the PLWH population potentially increasing arrhythmia risk (Heravi et al., 2019). TC smoking is also associated with abnormalities in VR as measured by QT interval and, in recent studies, the Tp‐e interval (Andrassy et al., 2003; Ileri et al., 2001; Ilgenli et al., 2015). Whereas the QT interval includes both ventricular depolarization and repolarization, and thus may lack the sensitivity and precision to detect subtle but important alterations in VR (Antzelevitch, 2007; Deyell et al., 2015; Dobson et al., 2013; Panikkath et al., 2011), the peak of the T wave to the end of the T wave (Tp‐e interval) represents only VR (Antzelevitch, 2001, 2007; Gupta et al., 2008), and prolongation of the Tp‐e interval has been shown to be a more reliable predictor of ventricular arrhythmias and sudden death than the QT interval in several clinical settings (Alizade et al., 2017; Antzelevitch, 2007; Castro Hevia et al., 2006; Deyell et al., 2015; Gupta et al., 2008; Kilicaslan et al., 2012; Lubinski et al., 1998; Panikkath et al., 2011; Shimizu et al., 2002; Tse et al., 2017; Yamaguchi et al., 2003). In fact, the Tp‐e interval is prolonged in many cohorts with increased risk of sudden death, including patients with congenital long QT syndrome, Brugada's syndrome, obstructive sleep apnea, coronary artery disease, hypertrophic cardiomyopathy, inducible ventricular arrhythmias, survivors of sudden cardiac death, and, importantly, in otherwise healthy people who smoke TCs (Alizade et al., 2017; Castro Hevia et al., 2006; Gupta et al., 2008; Ilgenli et al., 2015; Kilicaslan et al., 2012; Lubinski et al., 1998; Panikkath et al., 2011; Shimizu et al., 2002; Tasolar et al., 2014; Tse et al., 2017; Watanabe et al., 2004).

In our prior retrospective study, we reported a greater Tp‐e prolongation following acute TC smoking compared to EC use in the general population who smoke (Ip et al., 2020). In a prospective study comparing the acute effect of TCs and ECs on VR in the same individual, we found that ECs but not TCs significantly increased the Tp‐e interval following a provoked acute increase in heart rate (Ruedisueli et al., 2023). These findings are supported by preclinical studies in which acute exposure to aerosols generated from heating vegetable glycerin (VG) and/or propylene glycol (PG), both common EC liquid constituents, significantly prolonged ventricular repolarization in mice (Carll et al., 2022). Heated PG and VG rapidly degrade into aldehydes, such as acrolein. Heating of menthol‐flavored e‐liquid reportedly generates formaldehyde and acrolein at comparable levels to levels found in combusted tobacco smoke (Conklin et al., 2018; Ogunwale et al., 2017). In the current study in PLWH, neither acute TC smoking nor acute EC use resulted in significant changes in VR. Explanations for these disparate findings in PLWH compared to the general population remain unexplained but could be attributable to the HIV milieu or to ART, among other possibilities. Importantly, these findings underlie the urgency of conducting studies such as ours in the PLWH community, since it appears that acute EC and TC effects in PLWH may differ from the general population (Arastoo et al., 2020; Nguyen et al., 2024).

Within the PLWH study group, we recognized two distinct populations: PLWH who were currently using recreational drugs as evidenced by self‐report and confirmed by urine toxicology screen, and PLWH who were not. Initially we considered excluding PLWH who had a positive toxicology screen, but we realized that by doing so we would be excluding a group chronically understudied, those with behavioral health conditions, who also carry the heaviest smoking burden (Vuong et al., 2023). In our post‐hoc analysis of these subgroups, we found that at baseline, demographics were not different between the PLWH‐Drug and PLWH‐No Drug subgroups. Furthermore, while there were no differences in baseline hemodynamics and VR parameters, HRV parameters were consistent with significantly increased HF in the PLWH‐Drug group. Whether due to the high prevalence of cannabis use, which is associated with increased HRV (Schmid et al., 2010), or conversely, a rebound effect from recent stimulant use, or another explanation, this finding of increased vagal tone in PLWH who currently use recreational drugs is surprising and deserves further investigation. In the PLWH‐No Drug group, while most outcomes were not different after acute TC smoking compared to acute EC use, the effect of ECs on LF/HF was significantly less after using the EC compared to smoking the TC. Importantly, interpretation of this finding must also include the context that the acute increase in plasma nicotine was significantly smaller after acute EC use compared to acute TC smoking. Nonetheless, these findings may support a role for ECs as part of a harm reduction strategy in PLWH addicted to nicotine who do not use recreational drugs, especially if switching to ECs leads to differences in puffing behavior, and thus smaller increases in plasma nicotine. In contrast to the PLWH‐No Drug subgroup, in the PLWH‐Drug subgroup, there was no difference in any of our primary outcomes comparing acute TC smoking and EC use. We speculate that frequent use of recreational drugs known to impact the autonomic nervous system may obscure any expected benefits derived by switching from TCs to ECs.

## 6 LIMITATIONS

Despite many strengths of our study, we recognize several limitations. Most importantly, it is a small study, and the participants were a heterogeneous group, including a wide age range, and consequently, likely large differences in smoking duration. Although we initially enrolled PLWH between 21 and 45 years, enrollment was slow, and we were advised by two community advisory boards to increase the upper limits to 60 years to increase it. Plasma cotinine was measured to estimate recent tobacco exposure. Additionally, current viral loads, blood counts and ART was by self‐report, and could not be verified. Due to the small size, we were unable to perform meaningful analyses of these and other comorbidities. This limitation is offset by the study design in which each participant served as their own control and included over 100 experimental sessions (up to three per participant). It is unlikely that a significantly larger sample size would have changed the results, since our findings were almost never borderline. Nonetheless, this was a small, exploratory study that may have been underpowered for some outcomes. In addition to being a small study, we were able to enroll only one female. Despite working with two different community advisory boards for recruitment and study design, our efforts to recruit more females were unsuccessful. While this largely reflects the demographics of Americans living with HIV, further studies in females living with HIV are warranted. Although participants were instructed not to smoke within 12 h of the study session, at baseline, nicotine levels were elevated in four participants, likely indicative more recent use. Since our primary outcomes were related to the change in nicotine pre/post exposure, not the absolute final nicotine level, this smoking proximate to the study assumes less importance. Finally in the current study, we did not include a provocative maneuver to unmask any differences in VR as we did in our prior study comparing acute effects of TCs and ECs on VR in the general population (Ruedisueli et al., 2023). Future studies should include such a protocol.

In summary, while the acute hemodynamic, HRV, and VR effects of acute TC smoking compared to acute EC use were overall very similar in PLWH who smoke, there was some evidence to support a smaller shift towards sympathetic predominance after acute EC use compared to TC smoking. This relatively smaller increase in LF/HF was associated with a smaller increase in plasma nicotine following acute EC compared to TC use, and was most evident in PLWH who do not use recreational drugs. Since the acute effects of TC smoking and EC use on sympathetic activation do not necessarily mirror those of the general population, additional studies in this vulnerable population disproportionately affected by tobacco‐related health disparities are warranted. Finally, it is important to remember that although ECs may be less harmful than TCs, they are not harmless, and PLWH who switch from TCs to ECs should use them for the shortest duration possible.

## FUNDING INFORMATION

This work was supported by Tobacco Related Research Program (T31IP1813), NIH National Center for Advancing Translational Science (UCLA CTSI Grant Number L1TR001881), UCLA‐CDU CFAR Clinical Sciences Core and the UCLA‐CDU CFAR grant (NIH P30 AI152501), and the Sandler Foundation.

## CONFLICT OF INTEREST STATEMENT

None of the authors has any conflicts of interests.
